# 

**Published:** 1871-06

**Authors:** 


### CONTENTS.

COMMUNICATIONS.

Dental Education ..... 235
Address before the Graduating Class, in the Ohio College of Dental Surgery, March 1, 1871.... 243
May Fillings be made too dense? ..... 247
Phosphate of Lime ..... 250
Office Notes ..... 252
Demonstrations *vs.* Lectures ..... 253
Oxychloride of Zinc Treatment of Dental Pulps ..... 256
Remarkable Recovery of the Voice ..... 258

PROCEEDINGS OF SOCIETIES.

Minutes of the Wabash Valley Dental Association ..... 260

SELECTIONS.

Address before the Alumni Association ..... 264

EDITORIAL.

A New Impression Cup ..... 272
The Mad River Dental Society ..... 272
A Pumice Polisher ..... 273
The Northern Ohio Dental Association ..... 273
The Hot Air Syringe ..... 274
Eighth District Society of New York ..... 275
Progress ..... 276
Educational ..... 276
The Law ..... 279
In Memoriam—Dr. J. G. Willis ..... 280

